# Vitamin D Status Among Male Late Adolescents Living in Southern Switzerland: Role of Body Composition and Lifestyle

**DOI:** 10.3390/nu11112727

**Published:** 2019-11-11

**Authors:** Andrea Rabufetti, Gregorio P. Milani, Sebastiano A. G. Lava, Valeria Edefonti, Mario G. Bianchetti, Andreas Stettbacher, Franco Muggli, Giacomo Simonetti

**Affiliations:** 1 Istituto Pediatrico della Svizzera Italiana, 6500 Bellinzona, Switzerland; andrea.rabufetti@eoc.ch (A.R.); Giacomo.Simonetti@eoc.ch (G.S.); 2 Pediatric Unit, Fondazione IRCCS Ca’ Granda Ospedale Maggiore Policlinico, 20122 Milan, Italy; 3 Department of Clinical Sciences and Community Health, Università degli Studi di Milano, 20122 Milan, Italy; valeria.edefonti@unimi.it; 4 Pediatric Cardiology Unit, Department of Pediatrics, Centre Hospitalier Universitaire Vaudois (CHUV), and University of Lausanne, 1011 Lausanne, Switzerland; webmaster@sebastianolava.ch; 5 Faculty of Biomedical Sciences, Università della Svizzera Italiana, 6900 Lugano, Switzerland; mario.bianchetti@usi.ch; 6 Swiss Federal Department of Defence, 3000 Bern, Switzerland; andreas.stettbacher@vtg.admin.ch (A.S.);

**Keywords:** macronutrients, sunlight, physical activity, season, body composition

## Abstract

Background: Poor vitamin D status is a worldwide health problem. Yet, knowledge about vitamin D status among adolescents in Southern Europe is limited. This study investigated concentrations and modulating factors of vitamin D in a healthy population of male late adolescents living in Southern Switzerland. Methods: All apparently healthy subjects attending for the medical evaluation before the compulsory military service in Southern Switzerland during 2014-2016 were eligible. Dark-skin subjects, subjects on vitamin D supplementation or managed with diseases or drugs involved in vitamin D metabolism were excluded. Anthropometric measurements (body height, weight, fat percentage, mid-upper arm and waist circumference) and blood sampling for total 25-hydroxy-vitamin D, total cholesterol and ferritin concentrations testing, were collected. Participants filled in a structured questionnaire addressing their lifestyle. Characteristics of the subjects with adequate (≥50 nmol/L–≤250 nmol/L) and insufficient (<50 nmol/L) vitamin D values were compared by Kruskal-Wallis test or χ^2^ test. Odds ratios for 25-hydroxy-vitamin D insufficiency were calculated by univariate and AIC-selected multiple logistic regression models. Results: A total of 1045 subjects volunteered to participate in the study. Insufficient concentrations of vitamin D were detected in 184 (17%). The season of measurement was the most significant factor associated with vitamin D levels and approximately 40% of subjects presented insufficient vitamin D concentrations in winter. After model selection, body fat percentage, frequency and site of recreational physical activity, and the seasonality were significantly associated with the risk of vitamin D insufficiency. Conclusions: Among healthy male late adolescents in Southern Switzerland, about one every fourth subject presents a poor vitamin D status in non-summer seasons. Body fat percentage, frequent and outdoor recreational physical activity are modulating factors of vitamin D status in this population.

## 1. Introduction

There are two natural sources of vitamin D: food and especially ultraviolet B radiation on the skin [1]. A limited number of foods naturally contain vitamin D. Fish (mostly fatty fish), egg yolk and liver are good sources of vitamin D_3_. On the other hand, vitamin D_2_ is contained in various wild mushrooms [1,2]. Among European adolescents, the natural vitamin D intake is low except for countries such as Poland and Norway, which is attributed to high consumption of fish [3].

The amount of cutaneous vitamin D_3_ synthesis depends on a number of factors, including time spent outdoors, latitude, season, ethnicity and use of sunscreen [1]. Vitamin D synthesis occurs for about half the year in northern regions above approximately 35° latitude [3,4]. Unsurprisingly, therefore, lower-than desired concentrations of total 25-hydroxy vitamin D have often been detected, especially during the fall and winter months, in countries such Canada, Ireland, the United Kingdom and the northern United States [3,4]. It would be assumed that, in the sunniest areas of the world, this problem would be uncommon. However, in Australia, Brazil, India, Iran, Lebanon and Saudi Arabia many adolescents were found to have lower-than-desired concentrations of vitamin D [3,4].

Limited information is available on vitamin D status in adolescents living in Southern Europe. The objective of the present analysis was to obtain reliable and comparable data on vitamin D status from a large population of late adolescents living in Southern Switzerland, the sunniest region of this country (latitude 46°). The secondary aim was to investigate the role of a broad number of possibly relevant anthropometric, lifestyle and biochemical characteristics on vitamin D status in this population.

## 2. Methods

This investigation is part of the “CENERI study”, a cross sectional study in healthy male adolescents living in Southern Switzerland to investigate risk factors for chronic diseases later in life. In Switzerland, ostensibly male citizens between 18 and 19 years of age have to undertake a medical evaluation before the compulsory military service in the Army [5]. All apparently healthy subjects attending for the medical evaluation before the compulsory military service in Southern Switzerland from January 2014 to December 2016 were eligible for the “CENERI study”. Dark-skin subjects (Fitzpatrick skin phototype V or VI), subjects on supplementation with any form of vitamin D and subjects on treatment with anticonvulsant, glucocorticoid, antifungal, and anti-retroviral drugs or with any chronic endocrinologic or metabolic disease potentially affecting vitamin D metabolism, were excluded for the present analysis. Among the 4663 subjects who underwent the medical examination before the compulsory military service, 1045 (22%) Caucasians volunteered to participate in the study.

All measurements and data were collected in the same morning for each subject after an overnight fast. Beyond the routinely collected data on anthropometric measurements (body height and weight), participants were asked to answer a self-administered structured questionnaire addressing their main activity and lifestyle (especially recreational physical activity, smoking behavior and alcohol consumption). Body fat percentage, mid-upper arm and waist circumference were also measured. In addition, blood for total 25-hydroxy-vitamin D, total cholesterol and ferritin concentrations testing, was also collected.

Questions on lifestyle were structured as follows: (i) Frequency of recreational physical activity (never, 1 per week, 2–4 per week, 5–6 per week, every day), (ii) Duration of recreational physical activity session (≤1 h, >1–≤2 h, >2–≤3 h, >3 h), (iii) Site of recreational physical activity (indoor only, outdoor only, both indoor and outdoor), (iv) Frequency of alcohol consumption (never, 1 per week, 2 per week, 3–4 per week, 5–6 per week, every day), (v) Smoking (never, 1–10 cigarettes per day, 11–20 cigarettes per day, >20 cigarettes per day).

Subjects were weighed (wearing light clothes only) on a calibrated platform scale, with weight being rounded off to the nearest 0.1 kg. Standing height was measured barefooted to the nearest 0.1 cm. These measurements were used to calculate the body mass index. Mid-upper arm circumference was measured to the nearest 0.1 cm midway the acromion and the olecranon in the non-dominant arm. Waist circumference was measured to the nearest 0.5 cm with a non-stretching tape placed around the abdomen at the iliac crest. Body fat percentage was assessed by a validated bioimpedance analysis device (Omron^®^BF306, Omron Healthcare Europe BV, Hoofddorp, The Netherlands) [6]. After entering demographic and anthropometric data, the subjects were asked to remain in standing position while holding the hand-to-hand bioimpedance device by both hands and straightening both arms forward [7]. All demographics, anthropometric and lifestyle information were prospectively collected by a trained nurse.

An Abbott chemiluminescent microparticle immunoassay, which measures both 25-hydroxy vitamin D_2_ and 25-hydroxy vitamin D_3_, was applied for the determination of total 25-hydroxy vitamin D concentration in serum [8]. At an average total concentration of 49 nmol/L, 99 nmol/L and 187 nmol/L, the intra-assay coefficient of variation was 3.9%, 4.0%, and 4.0%, respectively. The corresponding inter-assay coefficient was 1.0%, 1.2%, and 2.6% [8]. Accuracy and reliability of the assay are assessed both in the Vitamin D Standardization Program [9] and in the Vitamin D External Quality Assessment Scheme [10]. The circulating levels of total cholesterol (enzyme assay) and ferritin (immunoassay) were measured in serum. All laboratory assessments were performed in the same accredited central laboratory (Viollier, Basel, Switzerland) using an Architect CI8200 (Abbott, Chicago, IL, USA) analyzer. The study was conducted in accordance with the Declaration of Helsinki, and the protocol was approved by the Ethics Committee of Southern Switzerland (RIF CE 2775). Informed written consent was obtained from all subjects to participate in the study.

### Data Analysis

Frequency distribution of continuous data were presented as median and interquartile range. Dichotomous data were presented as absolute and relative frequency. Concentrations of total 25-hydroxy-vitamin D were considered adequate if ≥50 nmol/L–≤250 nmol/L, insufficient if <50 nmol/L, deficient if <30 nmol/L or potentially toxic if >250 nmol/L [11]. Anthropometric, lifestyle and further laboratory characteristics of the subjects with adequate (50–250 nmol/L) and insufficient (<50 nmol/L) 25-hydroxy-vitamin D values were compared by Kruskal-Wallis test. χ^2^ test was used for comparing frequencies of categorical variables. The Bonferroni test adjustment for multiple comparisons was applied.

Odds ratios (ORs) of 25-hydroxy-vitamin D insufficiency and corresponding 95% confidence intervals (CI) from univariate logistic regression models were calculated for the following variables: age, body height, body weight, body mass index, body fat percentage, frequency/length and site of recreational physical activity, frequency of alcohol consumption, smoking, season (winter from 21 December; spring from 21 March, summer from 21 June and autumn from 21 September), cholesterol and ferritin concentrations. ORs of 25-hydroxy-vitamin D insufficiency and corresponding 95% CI were also derived from the best AIC-selected multiple logistic regression model including the following variables: age, body mass index, body fat percentage, waist circumference frequency/length and site of recreational physical activity, frequency of alcohol consumption, smoking, season, cholesterol and ferritin concentrations. In all analyses, significance was assumed if *p* < 0.05. Statistics was performed using the open source statistical language R, Vienna, version 3.5.3 (11 March, 2019).

## 3. Results

Body height (178.0 (173.5–182.0) vs. 177.5 (173.0–182.5) cm) and weight (72.2 (65.7–80.0) vs. 72.0 (65.0–80.5) kg) were similar in subjects who volunteered to participate in the study as compared with the remining 3618 subjects. Anthropometric, lifestyle and laboratory findings of the 1045 recruited subjects are given in Table 1. One hundred seventy-nine (17%) subjects presented with concentrations of total 25-hydroxy-vitamin D < 50 nmol/L. Among subjects with a concentration of vitamin D below 50 nmol/L, 24 (13%) had deficient levels of total 25-hydroxy-vitamin D. No subject presented with potentially toxic concentrations of the 25-hydroxy-vitamin D. The concentration of 25-hydroxy-vitamin D_2_ was always ≤5 nmol/L. The characteristics of the subjects with adequate or insufficient concentrations of 25-hydroxy-vitamin D are shown in Table 2. A total of 76 (7.2%) out of 1045 had a body mass index ≥ 30 kg/m^2^ and 34 (3.3%) ≤ 18.5 kg/m^2^.

**Figure 1:**
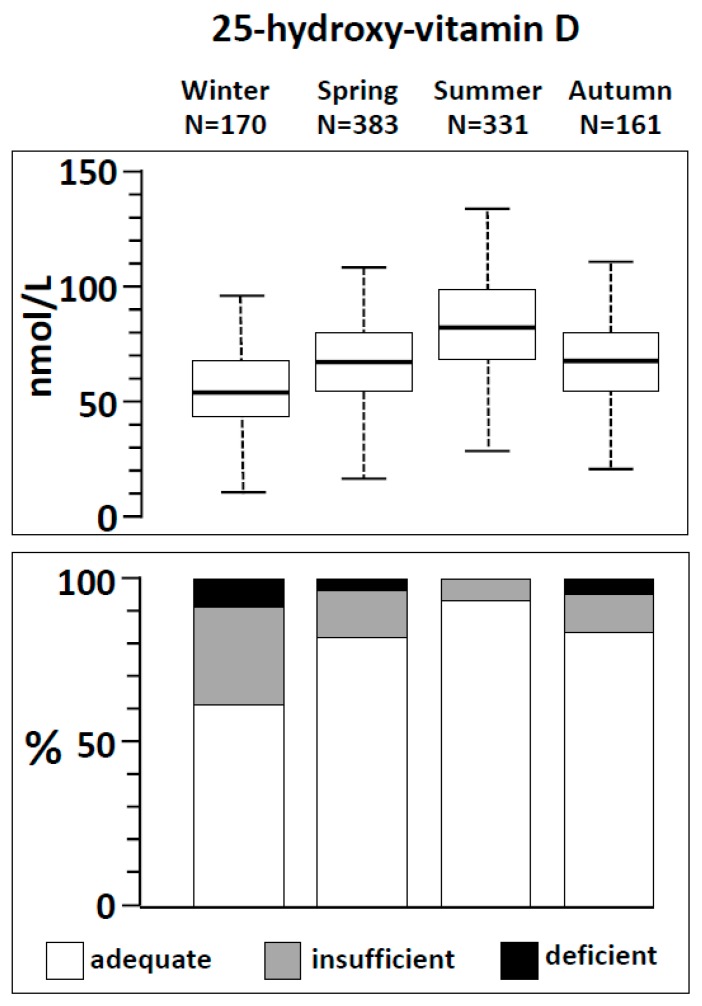
**Upper panel.** Box-plot of the circulating concentrations of 25-hydroxy-vitamin D across the four seasons. The boxes include values between the 1st and the 3rd quartile (i.e., the interquartile range). The whiskers include values between: 1st quartile − 1.5 times interquartile range and 3rd quartile + 1.5 times interquartile range. **Lower panel.** Frequency of adequate, insufficient or deficient concentrations of 25-hydroxy-vitamin D according to seasonality.

The season of measurement was the most significant factor associated with insufficient concentrations of 25-hydroxy-vitamin D. The concentrations vitamin D in the four seasons are depicted in Figure 1 (upper panel). Of note, 64 (38%) out of 170 subjects tested for 25-hydroxy-vitamin D level in winter presented insufficient concentrations of this vitamin, 70 (18%) out of 383 in spring, 18 (5.4%) out 331 in summer and 28 (17%) out of 161 in autumn. A total of 13 (7.6%) subjects in winter, 6 (1.6%) in spring and 5 (3.1%) in autumn, presented with deficient concentrations of 25-hydroxy-vitamin D. No subject had a deficient level of 25-hydroxy-vitamin D in summer (Figure 1, lower panel).

In the univariate logistic regression models (Table 3), body height (ORs 0.96, 95% CI 0.94–0.98), body mass index (OR 1.05, 95% CI 1.00–1.07, body fat percentage (OR 1.04, 95% CI 1.02–1.07), waist circumference (OR 1.02, 95% CI 1.00–1.03), the frequency of recreational physical activity 5–6 per week (OR 0.36, 95% CI 0.16–0.85), cigarettes consumption of 11–20 cigarettes per day (OR 0.41, 95% CI 0.22–0.77), the season (spring, OR 0.37, 95% CI 0.25–0.56, summer, OR 0.09, 95% CI 0.05–0.17, and autumn, OR 0.33, 95% CI 0.20–0.56) and cholesterol (OR 1.29, 95% CI 1.02–1.62) were positively (OR > 1) or inversely (OR < 1) associated with the risk of 25-hydroxy-vitamin D insufficiency.

Table 4 shows results from the multiple regression analysis. After model selection based on clinical plausibility and Akaike information criterion, the increase of body fat percentage was a significant risk factor (ORs >1) for 25-hydroxy-vitamin D insufficiency. A frequent (5–6 times per week) and outdoor physical activity and non-winter seasons were significant protective factors (ORs < 1) against 25-hydroxy-vitamin D insufficiency.

## 4. Discussion

This study points out that a large minority (17%) of healthy male late adolescents in Southern Switzerland, a region with a low natural vitamin D intake, has a poor vitamin D status. In this group of subjects, an increase in body fat percentage is a risk factor for vitamin D insufficiency. On the contrary, frequent and outdoor recreational physical activity and, especially, non-winter seasons are protective factors against vitamin D insufficiency. Yet, about one every fifth subject has insufficient concentrations of vitamin D also in spring and autumn.

The prevalence of hypovitaminosis D among adolescents from high-income countries largely varies among studies [12,13]. In the southeastern United States, vitamin D concentrations < 50 nmol/L were observed in about 4% of white male adolescents [14]. In the HELENA study, among 1006 subjects living in ten European countries, about 40% presented vitamin D concentration < 50 nmol/L [15]. Our data point out that insufficient concentrations of vitamin D are frequent among male late adolescents in Southern Switzerland and emphasize that vitamin D concentrations are strongly season dependent. Of note, the frequency of vitamin D insufficiency was very high in winter and still rather important (>15%) in autumn. A previous study suggests that in Ireland (latitude 51–55°) ultraviolet B radiation is effective for some vitamin D synthesis also in October [16]. On the other hand, very low doses were found in November and December. This study did not specifically investigate the ultraviolet B radiation in Southern Switzerland. However, the concentration of 25-hydroxy-vitamin D_2_ was always ≤5 nmol/L, confirming that also vitamin D of non-animal origin played a marginal role in vitamin D status in our population.

The peak bone mass is usually reached between 25 and 35 years of age and, in male subjects, is predicted by vitamin D status [17]. Hence, the years preceding the peak bone mass are considered as a critical period to maximize bone strength and maturation [18]. The European Academy of Pediatrics, the American Academy of Pediatrics and Endocrine guidelines currently do not routinely recommend supplying vitamin D in non-dark skinned, non-obese healthy adolescents or young adults [19,20,21]. The results of this study suggest that longitudinal studies should address the advantages of vitamin D supplementation in Caucasian late adolescents during winter. This finding is even more crucial for the bone metabolism, considering that only vitamins D concentrations >75 nmol/L have a clear-cut antifracture effect [22]. On the other hand, an increasing body of evidence highlights that vitamin D deficiency is associated with chronic and potentially life-threating conditions such as cardiovascular disease later in old adults and elderly [23]. In this study, about one every thirteen subjects had deficient concentrations of vitamin D in winter.

We found an association between vitamin D concentrations, frequent and outdoor physical recreational activity after adjusting for confounders. Although sun-light exposure does not occur exclusively during recreational physical activity, this finding confirms the beneficial role of outdoor activities. Yet, seasonal fluctuations of ultraviolet-B radiations might decrease the effects of sun-light exposure during non-summer seasons and especially in winter [22,23]. Differently from previous observations [24,25], this study did not identify any association between vitamin D and a marker of inflammation such as ferritin. This might be due to the fact that the population of this study exclusively included healthy late adolescents without any chronic disease. Also, we did not find any association with cholesterol or alcohol consumption. A possible explanation is that most prior studies have included subjects with a much broader range of age and the mentioned factors could become determinant when persisting for long-term periods [26]. Previous studies found body mass index to be inversely associated with vitamin D concentrations. However, residual confounding such as physical activity or body composition assessment have not always been considered [27]. Furthermore, body mass index cannot distinguish lean from fat mass, especially in youth [28]. One of the advantages of this study is that many anthropometric characteristics were explored disclosing an association between body fat percentage and vitamin D concentrations levels after adjusting for confounders. A tendency to accumulate vitamin D (a liposoluble compound) in fat depots or an impaired vitamin D intestinal absorption or hydroxylation in adipose tissue are likely to underly this association [29]. Of note, some studies have also hypothesized that vitamin D insufficiency itself could reduce weight loss or favoring weight gain [29].

This study has many strengths and limitations. The main strengths are the large number of apparently healthy subjects enrolled with a narrow range of age and the concomitant determination of many potential modulators of vitamin D including the body fat percentage. Furthermore, Southern Switzerland is considered the sunniest region of Switzerland: therefore, it is possible that the number of late adolescents with vitamin D insufficiency might be even higher in the other parts of Switzerland. The main limitation of this study is the exclusion of females. Second, results are partly based on self-reports, which might not always correspond to the actual behavior of the responders. Third, its cross-sectional nature prevents to longitudinally evaluate vitamin D concentrations throughout the seasons. Fourth, we did not analyze some common inflammatory indices, such as C-reactive protein. Finally, the use of sunscreen, which is usually not very common among male adolescents and young adults in Switzerland [30], was not investigated.

## 5. Conclusions

This study showed that about one every fourth healthy male late adolescent in Southern Switzerland presents insufficient concentrations of vitamin D during non-summer seasons. Low body fat and both frequent and outdoor recreational physical activity are associated with adequate vitamin D level this population.

## Figures and Tables

**Table 1:** Baseline characteristics of the enrolled subjects. Data are given as absolute frequency (and percentage) or median (and interquartile range).

N	1045
**Main activity**	
Student	433 (41)
Worker	612 (59)
**Anthropometric characteristics**	
Body height, cm	178.0 (173.5–182.0)
Body weight, kg	72.2 (65.7–80.0)
Body mass index, kg/m^2^	23.0 (21.0–25.1)
Body fat percentage, %	17.8 (13.7–23.1)
Mid-upper arm circumference, cm	27.0 (25.0–30.0)
Waist circumference, cm	80 (75–87)
**Frequency of recreational physical activity**	
Never	219 (21)
1 per week	174 (17)
2–4 per week	472 (45)
5–6 per week	88 (8.4)
Every day	92 (8.8)
**Length of recreational physical activity session**	
≤1 h	66 (8.0)
>1–≤2 h	602 (73)
>2–≤3 h	143 (17)
>3 h	15 (1.8)
**Site of recreational physical activity**	
Indoor (only)	148 (18)
Outdoor (only)	340 (41)
Both indoor and outdoor	338 (41)
**Frequency of alcohol consumption**	
Never	201 (19)
1 per week	393 (38)
2 per week	285 (27)
3–4 per week	112 (11)
5–6 per week	42 (4.0)
Every day	12 (1.2)
**Smoking**	
Never	602 (58)
1–10 cigarettes per day	299 (29)
11–20 cigarettes per day	130 (12)
>20 cigarettes per day	14 (1.3)
**Biochemical indices**	
Total 25-hydroxy-vitamin D, nmol/L	68 (55–82)
Cholesterol, mmol/L	3.9 (3.5–4.3)
Ferritin, µmol/L	76.0 (52–109)

**Table 2:** Characteristics of subjects with adequate and insufficient circulating 25-hydroxy-vitamin D. All variables were non-normally distributed. Data are given as absolute frequency (and percentage) or median (and interquartile range). The Kruskal-Wallis test was used for continuous variables. Chi-squared test was used for categorical variables. The Bonferroni test adjustment was applied to account for multiple comparisons.

Subjects Characteristics	25-hydroxy-vitamin D		
	Adequate (≥50 nmol/L)	Insufficient (<50 nmol/L)	*p*-value
**N**	866 (83)	179 (17)	
**Main activity**			
Student	356 (41)	77 (43)	0.7
Worker	510 (59)	102 (67)	
**Anthropometric characteristics**			
Body height, cm	178.0 (173.0–182.0)	176.0 (171.5–180.0)	0.003 **
Body weight, kg	72.5 (65.9–80.0)	71.3 (64.9–82.0)	0.9
Body mass index, kg/m^2^	22.9 (21.0–25.0)	23.0 (20.7–26.3)	0.3
Body fat percentage, %	17.4 (13.4–22.7)	20.1 (14.6–25.2)	<0.0001 ***
Mid-upper arm circumference, cm	27.0 (25.0–29.5)	27.0 (24.0–30.1)	0.4
Waist circumference, cm	80 (75–86)	80 (75–91)	0.1
**Frequency of recreational physical activity**			
Never	177 (20)	42 (24)	0.03 *
1 per week	134 (16)	40 (22)	
2–4 per week	396 (46)	76 (43)	
5–6 per week	81 (9.4)	7 (3.9)	
Every day	78 (9.0)	14 (7.8)	
**Duration of recreational physical activity session**			
≤1 h	50 (7.3)	16 (12)	0.3
>1–≤2 h	509 (74)	93 (68)	
>2–≤3 h	118 (17)	25 (18)	
>3 h	12 (1.7)	3 (2.2)	
**Site of recreational physical activity**			
Indoor (only)	117 (17)	31 (23)	0.1
Outdoor (only)	281 (41)	59 (43)	
Both indoor and outdoor	289 (42)	47 (34)	
**Frequency of alcohol consumption**			
Never	161 (19)	40 (22)	0.1
1 per week	338 (39)	55 (31)	
2 per week	224 (26)	61 (34)	
3–4 per week	97 (11)	15 (8.4)	
5–6 per week	35 (4.0)	7 (3.9)	
Every day	11 (1.3)	1 (0.6)	
**Smoking**			
Never	483 (56)	119 (67)	0.018 *
1–10 cigarettes per day	253 (29)	46 (26)	
11–20 cigarettes per day	118 (14)	12 (6.7)	
>20 cigarettes per day	12 (1.4)	2 (1.1)	
**Season of measurement**			
Winter	106 (12)	64 (36)	<0.0001 ***
Spring	313 (36)	70 (39)	
Summer	313 (36)	18 (10)	
Autumn	134 (16)	27 (15)	
**Biochemical indices**			
Cholesterol, mmol/L	3.9 (3.5–4.3)	4.0 (3.6–4.4)	0.049 *
Ferritin, µmol/L	76.0 (52.0–109.0)	76.5 (51.0–103.5)	0.9

* *p* < 0.05, ** *p* < 0.01, *** *p* < 0.0001.

**Table 3:** Odds ratios (ORs) of 25-hydroxy-vitamin D insufficiency and corresponding 95% confidence intervals (CIs) from univariate logistic regression models.

Subject Characteristic	OR	Lower 95%CI	Upper 95%CI	*p*-Value
**Body height, cm**	0.96	0.94	0.98	0.004 **
**Body weight, kg**	1.01	0.99	1.20	0.4
**Body mass index, kg/m^2^**	1.05	1.00	1.07	0.03 *
**Body fat percentage, %**	1.04	1.02	1.07	<0.0001 ***
**Mid-upper arm circumference, cm**	0.99	0.95	1.04	0.09
**Waist circumference, cm**	1.02	1.00	1.03	0.03 *
**Frequency of recreational physical activity**				
Never	reference			
1 per week	1.26	0.77	2.05	0.4
2–4 per week	0.81	0.53	1.23	0.3
5–6 per week	0.36	0.16	0.85	0.02 *
Every day	0.76	0.39	1.46	0.5
**Duration of recreational physical activity session**				
≤1 h	reference			
>1–≤2 h	0.57	0.31	1.05	0.06
>2–≤3 h	0.66	0.33	1.35	0.3
>3 h	0.78	0.19	3.12	0.7
**Site of recreational physical activity**				
Indoor	reference			
Both indoor and outdoor	0.79	0.49	1.29	0.4
Outdoor (only)	0.61	0.37	1.01	0.05
**Frequency of alcohol consumption**				
Never	reference			
1 per week	0.66	0.42	1.03	0.06
2 per week	1.10	0.70	1.71	0.7
3–4 per week	0.62	0.33	1.19	0.1
5–6 per week	0.80	0.33	1.95	0.6
Every day	0.37	0.05	2.92	0.3
**Smoking**				
Never	reference			
1–10 cigarettes per day	0.74	0.51	1.07	0.1
11–20 cigarettes per day	0.41	0.22	0.77	0.006 **
>20 cigarettes per day	0.68	0.15	3.06	0.6
**Season of measurement**				
Winter	reference			
Spring	0.37	0.25	0.56	<0.0001 ***
Summer	0.09	0.05	0.17	<0.0001 ***
Autumn	0.33	0.20	0.56	<0.0001 ***
**Biochemical indices**				
Cholesterol, mmol/L	1.29	1.02	1.62	0.03 *
Ferritin, µmol/L	1.0	0.99	1.01	0.9

* *p* < 0.05, ** *p* < 0.01, *** *p* < 0.0001.

**Table 4:** Odds ratios (ORs) of 25-hydroxy-vitamin D insufficiency and corresponding 95% confidence intervals (CIs) as derived from the best Akaike information criterion-selected multiple logistic regression model. The original model included the following variables: body mass index, body fat percentage, mid-upper arm circumference, waist circumference, frequency of recreational physical activity, duration of recreational physical activity session, site of recreational physical activity, season, frequency of alcohol consumption, smoking, cholesterol and ferritin.

Subject Characteristic	OR	Lower 95%CI	Upper 95%CI	*p*-Value
**Body fat percentage, %**	1.04	1.01	1.07	0.01 *
**Frequency of recreational physical activity**				
2–4 per week	0.64	0.40	1.02	0.6
5–6 per week	0.32	0.13	0.78	0.01 *
Every day	0.64	0.31	1.35	0.2
**Site of recreational physical activity**				
Both indoor and outdoor	0.80	0.47	1.37	0.4
Outdoor (only)	0.56	0.33	0.96	0.04 *
**Season of measurement**				
Spring	0.31	0.19	0.49	<0.0001 ***
Summer	0.08	0.04	0.17	<0.0001 ***
Autumn	0.24	0.13	0.45	<0.0001 ***
**Biochemical indices**				
Cholesterol, mmol/L	1.25	0.95	1.66	0.1

* *p* < 0.05, *** *p* < 0.0001.
